# Chromosomal translocations highlighted in Primitive Neuroectodermal Tumors (PNET) and Ewing sarcoma

**Published:** 2014

**Authors:** IO Trancău

**Affiliations:** * Foişor” Orthopedics Clinical Hospital, Bucharest, Romania; “Carol Davila” University of Medicine and Pharmacy, Bucharest, Romania

**Keywords:** chromosomal translocations, Primitive Neuroectodermal Tumors (PNET), Ewing sarcoma

## Abstract

Almost 200 molecular markers in oncology, very important in the diagnosis, prognostic and treatment were identified.

The cell and tissue markers and also the circulating (sanguine) ones are genetic markers of the hereditary and non-hereditary tumors.

Also extremely important are the regulatory ways of cell growth and differentiation, of the cell “senescence” and cell death (apoptosis).

The term of “tumor marker” concerns a variety of molecules or processes that are different in the normal cell compared with the malign cell.

The tumor markers may include modifications to the genetic level (mutations, deletions or genes amplifications) to the transcription level (super expression or sub-expression), to the translation level (high or low quantities of proteins, abnormal glycosylation of proteins) and/or to the functional level (the level of cell differentiation or presence of neo-vascularisation).

Cancer is a genetic disease. There is a deregulation at the genes level that controls the cell division and withdrawal from the cell cycle or there is a genetic susceptibility.

In other words, cancer is an end point for several phases in which the oncogenes and stimulatory signals and inhibitors produced and controlled by the products of these oncogenes are involved.

## Introduction

Almost 200 molecular markers in oncology, very important in the diagnosis, prognostic and treatment were identified. 

The cell and tissue markers and also the circulating (sanguine) ones are genetic markers of the hereditary and non-hereditary tumors. 

Also extremely important are the regulatory ways of cell growth and differentiation, of the cell “senescence” and cell death (apoptosis). 

More than 90% of the human cancers are developed to the level of the epithelial cells (carcinoma) and most of times they have the surface epithelia (tegument, breathing pipe, intestinal tube) or secondary sexual organs (prostates, breast) as a starting point. Thus, there is a link between the carcinogenic agents that cannot go too far in the organism and the tissues with a higher rate of cell proliferation or endocrine controlled tissues. 

The term of “tumor marker” concerns a variety of molecules or processes that are different in the normal cell compared with the malign cell.

The tumor markers may include modifications to the genetic level (mutations, deletions or genes amplifications) to the transcription level (super expression or sub-expression), to the translation level (high or low quantities of proteins, abnormal glycosylation of proteins) and/or to the functional level (the level of cell differentiation or presence of neo-vascularisation).

Each of these modifications can be assessed through one or several methods of research.

Cancer is a genetic disease. There is a deregulation at the genes level that controls the cell division and withdrawal from the cell cycle or there is a genetic susceptibility. 

In other words, cancer is an end point for several phases in which the oncogenes and stimulatory signals and inhibitors produced and controlled by the products of these oncogenes are involved. 

The genetic alteration can dominantly affect the proto-oncogene, which becomes active and determines positive signals for cell proliferation and the modification can be recessive affect the suppressing tumor gene, this leading to a negative regulation of the cell division. 

The mutations at the level of the tumor suppressing genes can have a dominant-negative effect, by the producing modified protein that enters in competition with the normal one, obstructing its activity (an example is P53 protein, controlled by the suppressing gene TP53). 

The second genetic cause of neoplasia is represented by the genetic susceptibility (8-10% of the cases), inherited and always affecting the tumor suppressing genes. These have a different penetration and determine the genetic risk to develop a neoplasia. 

All these genes produce molecular markers that have a diagnosis value and create the genetic profile of the neoplasia cells. At the same time, these markers are used for the prognostic evaluation and therapeutic efficiency. The mutations at the genes’ level are punctiform mutations, deletions, or inadequate expressions of genes [**1**].

**The genetic markers** are grouped in:

1. Tumor markers associated with the modifications at the genes’ level (TP53, RAS, HER-2/neu, RET, BCL2, BCL1-PRAD1-CCND1, REL, MYC, BCL6, p16-INK4a-CDKN2A). 

2. Tumor markers derived from chromosomal instability (loss of the heterozygote state, instability of “micro-satellites”) 

3. Tumor markers derived from non-spontaneous chromosomal anomalies (non-random): hematopoietic tumor and activation of proto-oncogenes – BCL2, BCL1, c-myc, BCL6, TAL1 – hematopoietic tumors and trans-membrane proteins – NPM-ALK, BCR-ABL t(9;22) (q34, q11), PML-RARA (t15; 17) (q22; q12), AML-ETO t(8, 21) (q22; q22), translocations emphasized to the level of the primitive neuroectodermal tumors – (PNET) – Ewing Sarcoma EWS/FLI 1 t(11, 22) (q24, q12), EWS-ERG t(21; 22) (q22; q12), EWS-ETV1 t(7; 22) (p22; q12), EWS-fev t(2; 22) (q33; q12), EWS-E1AF t (17; 22) (q 12; q12), EWS-ATF1 t(12; 22) (q13; q12), EWS-WT1 t(11; 22) (p13; q12), EWS-CHN t(9; 22) (q22-31; q11-12), EWS-CHOP t(12; 22) (q13; q12), FUS-CHOP t(12; 16) (q13; p11), EWS/FLI t(11; 22) (q24; q12), PAX3-FKHR t(2; 13) (q35; q14) and PAX7-FKHR t(1; 13) (p36; q14), SYT-SSX t(x; 18) (p11.2; q11.2), PDGFB-COL1A1 t(17; 22) (q22; q13); epithelial tumors – RET NTRK1

4. Markers related to clonation (clone markers): inactivity of chromosome X, determining the heavy chains of immunoglobulin (igH), modification of receptors at the level of T lymphocytes (rearrangement of the genes controlling the synthesis of receptors from the level of T lymphocytes – TCR genes). 

The specific translocations were initially identified in hematological tumors, and then these were also proved at the level of some solid neoplasm. 

The chromosomal translocations have two important consequences in the hematopoietic tumors: the superposition of a proto-oncogene on the gene controlling the receptors for the T lymphocyte or on the gene controlling the synthesis of immunoglobulins (Ig), inducing the activation of oncogenesis and request of a fusion gene that codifies a chimera protein. These genes control the transcription factors, and the break within the transcription control plays a major role in oncogenesis. 

The activation of the following proto-oncogenes: BCL2, BCL1, c-myc, BCL6, TAL1 (acute leukemia with T cells) is produced in hematopoietic tumors.

Sarcomas were the solid tumors studied from the cytogenetic perspective (of translocations) [**2**]. 

The following chromosomal translocations were emphasized at the level of the primitive neuroectodermal tumors (PNET – primitive neuroectodermal tumors) and Ewing sarcoma (ES).

**EWS-FL1 1, t(11, 22) (q24; q12)**

The analysis of the cariotip revealed a chromosomal translocation t(11, 22) (q24; q12) specific for a tumor – Ewing sarcoma type, or of primitive neuroectodermal tumors (PNET) type (in 86% from the above mentioned cases). 

The translocation (11; 22) resulted from the fusion of the N – terminal region of the EWS gene, rich in residues of glutamine, serine and tyrosine with the DNA connection field of ETS that is held on its turn by the FLI 1 gene (friend leukemia integration site 1) [**3**], known as proto-oncogene FLI 1. 

EWS is an expression of the gene, located on the 22nd chromosome that codifies the connection protein of ARN. FLI 1 is a gene located on the 11th chromosome, being a member of the ETS family, as transcription factors (ETS – E twenty-six – family of transcription factors). 

The oncogene effect of translocation t(11; 22) is caused by the formation of a chimera protein, meaning a protein formed by the fusion of several codified proteins by uniting two or several genes that would naturally codify the separate proteins. 

This fusion protein has the potential of promoting the tumor-genesis, activating the transcription of the aberrant factor transcription [**4**], this being different from the functional perspective compared with the normal version of FLI 1 [**5**].

Several types of EWS/FLI 1 fusion were studied, of which two main types, namely: 

- fusion of exon 7 from EWS with exon 6 from FLI 1 (type 1)

- fusion of exon 7 from EWS with exon 5 from FLI 1 (type 2) 

The 1st and 2nd type represent 85% of the fusions between EWS – FLI 1 [**6**]. 

The 1st type of EWS/FLI 1 fusion is considered a positive factor of prediction for survival [**7**].

The molecular detection of translocation t(11; 22) is valuable in the differential diagnosis of the tumors with small cells – round and in stadialization and prognostic of Ewing sarcomas [**8**].

From the EWS – FLI 1 translocation perspective, the positive cells have amplifications of RT – PCR in the hematogenous bone marrow and peripheral blood, for patients with metastasis and also for those without metastasis suffering from Ewing sarcomas and primitive neuroectodermal tumors [**9**]. 

**EWS – ERG, t(21; 22) (q22; q12)**

T(21; 22) is a version of the translocation of EWS gene, present in 5% of the cases of Ewing sarcoma. 

ERG gene is located on the chromosome 21.

ERG (ETS related gene) is a member of the ETS family of the transcription factors.

The ERG – ETS fusion can be found in the EWS gene that determines the Ewing sarcoma.

The family of ETS transcription factors is involved in cell differentiation, control of cell cycle, cell migration, cell proliferation, apoptosis (programmed cell death) and angiogenesis. 

The ERG transcription factor, codified by the ERG gene is a nuclear protein that relates sequences rich in purines. 

The ERG (DNA) protein fusions with the EWS (ARN) proteins, and this fusion, function as activators of the transcription. 

ERG and the EWS-ERG fusion proteins inhibit the apoptosis (hybrid expression EWS/ERG protein) [**10**].

**EWS-ETV1, t(7; 22) (p22; q12)**

The ETS translocation variant 1 is a protein codified in humans by gene ETV1. 

This version of chromosomal translocation is rare and was identified in two cases of PNET (primitive neuroectodermal tumors) [**11**,**12**].

All the ETS proteins (E – twenty six) contain a connection field of the DNA that connects the DNA sequences that contain 5’ – CGGA[AT]-3’. 

The protein codified by this gene (ETV) contains a short domain of acid transactivation (transactivation domain – TAD) in the N-terminal area, outside the DNA connection domain of ETS in the C-terminal region. 

Therefore, identical sequences of nucleotides were found in the most chimeric EWS/FLI 1 and EWS-ERG transcripts [**13**].

This gene is involved in the chromosomal translocations that determine multiple fusion proteins, including the EWS – ETV1 in Ewing Sarcoma, prostate cancer, melanomas, stromal gastro-intestinal tumors.

The transcript activator that relates the DNA sequences includes the pentanucleotide 5’ – CGGA [AT]-3 consensual sequences. 

**EWS-fev, t(2; 22) (q33; q12)**

The FEV gene is located on chromosome 2 and contains 3 exons. 

It is part of the ETS family of the transcript factors along with EWS/FLI 1 (90-95%), EWS-ERG (5-10%), being a new member and extremely rare in this family. 

This gene codifies a protein of 238 amino acids that has a DNA connection domain similar with the one of FLI 1 and ERG genes. 

Unlike FLI 1 and ERG, FEV loses the regulatory domain of transcription at the N-terminal end.

The C-terminal end of FEV is rich in alanine, which suggests a potentially repressing activity of the transcription.

The FEV expression is detected in the prostates adult tissues and of the thin intestine, so there is a predilection of the extra-skeleton localization [**14**].

**EWS-E1AF, t(17; 22) (q12; q12)**

The chromosomal translocation t(17; 22) leads to the fusion of EWS genes with E1AF and was described in a non-differentiated sarcoma of a child [**15**]. 

E1AF is a new member of the family of ETS genes (of the transcription factors), and, is located on chromosome 17q21 and codifies an adenovirus E1A enhancer-binding protein.

As in the case of the other fusion proteins, specific for the family of Ewing sarcomas, it is assumed that the ARN binding domain of EWS can be changed with the DNA binding domain of E1AF. 

**EWS – ATF1, t(12; 22) (q13; q12)**

ATF1 is a gene that codifies a protein called ATF1 transcription factor, depending on AMPc. 

The EWS-ATF1 translocation is frequent and specific for the malign melanoma of soft tissues (sarcoma with clear cells). 

EWS-ATF1 fusion protein (chimeric protein) resulted from the binding of the N-terminal domain of EWS of βZIP binding DNA domain of ATF1 and possibly, the activation of the target genes ATF1 (signal genes) [**16**].

**EWS-WT1, t(11; 22) (p13; q12)**

This repetitive translocation associated with the desmoplastic sarcoma with small round cells results from the fusion of the EWS gene with the gene of the Wilms tumor on the chromosome 11p13.

WT1 gene codifies a transcription factor that contains four marked domains or that contain zinc-finger at the C-terminal end and to the N-terminal end, rich in proline / glutamine at the level of the DNA binding domain. 

This transcription factor has a very important role in the normal development of the genital – urinary apparatus [**17**].

WT1 is a suppressing oncogene, specifically inactivated in a subset of Wilms tumors, and the mutations were found at the level of the cell enhancing line of the suspect individuals [**18**]. 

The rupture point in the EWS-WT1 translocation is located at the level of the intron, between exons 7 and 8 of EWS and intron between exons 7 and 8 or WT1, resulting a fusion of the functional domains of the two genes. 

The fusion protein (chimeric) includes the N-terminal domain of EWS and the DNA binding domain of WT1, marked by zinc. This protein modulates the transcription at the level of the target places of WT1 [**19**].

**EWS-CHN, t(9;22) (q22-31; q11-12)**

This repetitive translocation was observed in the extra-skeleton myxoid chondrosarcoma [**20**].

NOR1 (neuron-derived orphan receptor 1) known as NR4A3 (sub-family 4 of the nuclear receptor, group A, member 3) is a protein codified by NR4A3 gene to human [**21**].

NOR1 is a member of the family of nuclear receptors held by the intra-cellular factors of transcription and plays an important role in the proliferation, differentiation, metabolism and cell apoptosis [**22**].

CHN is a human gene, homologue of gene NOR1 from mouse (chitinases class I – CHN), this gene controlling an orphan nuclear receptor that has the DNA binding domain zinc-finger and it is located on 9q 22-31 [**23**].

The chimeric gene (of fusion) EWS – CHN codifies a fusion protein to which the ARN binding domain from the C-terminal end of EWS is replaced with the CHN protein, including a high N-terminal domain, a central binding DNA domain and a C-terminal domain of binding / dimerization [**24**].

**EWS- CHOP t(12; 22) (q13;q12)**

t(12;22) was described in the myxoid liposarcoma with round cells (the most common sub-type from the liposarcomas category) [**25**]. Liposarcoma is a tumor of soft tissues, sub-classified in four histological forms: well differentiated liposarcoma, myxoid liposarcoma with round cells, pleomorphic liposarcoma, to be differentiated (non-differentiated) liposarcoma.

CHOP (C/EBP enhancer binding protein – homologue protein) is a nuclear protein that was identified as a dominantly negative transcript inhibitor factor from the C/EBP and LAP factor class.

This protein was also named DDIT3 (DNA damage – inducible transcript 3’) and GADD153 (growth arrest and DNA damage inducible gene). Isolation DDIT3 suffers rearrangements in the myxoid liposarcoma, characteristic for translocation t(12; 16) (q13; p11). The DDIT3 gene fusions with a gene from the 16 chromosome, called FUS [**26**,**27**]. 

EWS-CHOP leads to a fusion between the N-terminal end of EWS gene and CHOP gene, which has as a result a fusion gene (chimeric gene).

CHOP is located on chromosome 12q13. 

At the molecular level, the punctiform mutation appears in the 7th intron, close to the ALU sequence for EWS gene and similarly, the modification (mutation) at the level of the CHOP gene appears in the intron 1, also close to ALU sequence [**28**]. 

The presence of EWS-CHOP fusion gene in the myxoid liposarcoma with the round cells indicates that the N-terminal group of the FUS gene can be replaced by the N-terminal group of EWS gene, which has as a result a CHOP protein (oncoprotein) fusion and the fact that the two N-terminal groups, when the fusion and result highlight certain transcription factors, have a similar oncogene potential. 

**FUS-CHOP, t(12; 16) (q13; p11)**

T(12; 16) (q13; p11) is a translocation specific for the myxoid liposarcoma with round cells [**29**],. this chromosomal anomaly results following the fusion of a gene from the 16th chromosome, called FUS/TLS (fused in sarcoma / translocated in liposarcoma) with a gene from the 12th chromosome, called CHOP, that codifies a nuclear protein that is an inhibitor transcription factor, dominantly negative, from the factor class C/EBP – enhancer binding protein and LAP (latency associated peptide) – see translocation EWS – CHOP t(12, 22) (q13; q12). 

The FUS product contains a rich QSY segment and an ARN binding domain, similar with the protein of EWS gene. After the rearrangements, the so-called ARN binding domain of FUS is replaced by the entire tail region of CHOP gene that contains the basic domain, based on the “zipper” motif of leucynes or leucynes zippers. 

The “leucyne zippers” motif consists of a region (area) formed by amino acids in which each seventh amino acid is a leucyne.

As in the fusion of the EWS gene, the domain of FUS gene provides an activating domain for the transcription towards a presumptive binding domain of the DNA related to CHOP gene [**30**].

**PAX 3 – FKHR, t(2; 13) (q35; q14) and PAX 7 – FKHR, T(1; 13) (P36; Q14)**

FKHAR (FORKHEAD Drosophila homologue 1 rhabdomyosarcoma is located at the 13q14.1 level.

PAX (paired box) is part of the family of the transcription factors that contain a pair field and usually, a partial or integral homeo-domain. 

PAX 3 was identified with the embryological development of the ear, eye and facial development. To human, this gene transcribes a protein that contains 479 amino acids. PAX 3 is frequently expressed in melanomas and contributes to the survival of the tumor cells [**31**]. 

PAX 7 is probably associated with the myogenesis. To human, the protein transcribed by this gene has 520 amino acids. 

PAX 7 is involved in the post-natal development, being involved in the propagation of certain satellite myogenetic cells, but without specification [**32**]. 

FKHR is frequently translocated with PAX 3 and PAX 7 genes in the alveolar rhabdomyosarcoma [**33**]. 

The fusion between FKHR gene and the control genes of the PAX 3 or PAX 7 embryological development is produced at the 13p14 level for FKHR gene and 2p5 level for gene PAX 3, respectively level 13q14 for PAX 7 gene [**34**]. 

PAX 3 and PAX 7 codify a transcription factor, whose DNA binding domain controls the embryological development, activating certain specific target genes. 

Following the PAX/FKHR translocation, the fusion protein contains the DNA binding domain, the DNA binding domain of FKHR, reduced in the C-terminal region of FKHR [**35**].

**SYT-SSX (t(x; 18) (p11.2; q11.2)**

SYT-SSX fusion gene results in following the chromosomal translocation t(x; 18) (p11; q11) and is present in over 80% of the synovial sarcomas. As a result of this translocation, the SYT gene from the 18th chromosome fusions with SSX1, or with SSX2, at the level of Xp11.2 (highly homologue genes). 

SYT-SSX 1 or SYT-SSX2 fusion protein present at the C-terminal end of Syt, amino acids replaced by the amino acids of SSX proteins from their C-terminal end [**36**]. The fusion protein functions as aberrant transcript factors [**37**]. 

There is a relation between the synovial sarcoma sub-types (monophasic vs. biphasic) and the implication of SSX 1 or SSX 2 genes. 

- the SYT-SSX 1 translocations in biphasic sarcomas 

- the SYT-SSX 2 translocations in monophasic synovial sarcomas.

The patients with SYT-SSX 2 had a longer free interval until the apparition of metastasis unlike the SYT-SSX 1 patients, for whom the metastasis appeared faster [**38**].

**PDGFB-COL1A1, t(17; 22) (q22, q13**) 

This chromosomal translocation was identified in the protuberant dermatofibrosarcoma, a tegument tumor of intermediary malignity.

The juvenile form of this tumor, called fibroblast with giant cells has as characteristic, from the cytogenetic perspective, the presence of supra-numbered ring chromosomes derived from the translocation t(17; 22) [**39**,**40**]. 

A fusion at the genes level that codifies a protein from the family of the growth factors derived from platelet (PDGF – platelet – derived growth factor) and the gene that controls the collagen synthesis 1α1 (COL 1A1) is produced [**41**]. 

The proto-oncogene PDGFβ, C-sis form suffers modifications of its activity and has a mutagen action for many cell types (C-sis is oncogene derived from PDGF) [**42**]. 

COL 1A1 is a major constituent of the conjunctive tissue matrix. This type of collagen (collagen type 1) is the most spread and can be found in cartilage, bone, tendon, skin, sclera. 

The COL 1A1 gene is located on the 17th chromosome to position 21.33 (17p21.33). 

The PDGF gene is located on the 22nd chromosome to position 13.1 (22q13.1). 

The genetic fusion leads to the deletion of exon 1, from the PDGFβ level, and so to the modification of the normal regulation of the grown factor release [**43**].

Transfection of the DNA of the cells affected in the protuberant dermatofibrosarcoma in cultures of fibroblast cells (NIH3T3) highlights the chimeric section (of fusion) of COL 1A1/ PDGFβ, whose activity is modified [**44**].

## Conclusion

Knowing the activation idea of the PDGFβ gene, and also of the substances that block certain phases from the activation of this gene, a specific therapy for the protuberant dermatofibrosarcoma is prefigured.
